# Supplementary material for: A UMLS-based spell checker for natural language processing in vaccine safety
Source: BMC Med Inform Decis Mak. 2007 Feb 12;7:3. doi: 10.1186/1472-6947-7-3 (PMC1805499; doi:10.1186/1472-6947-7-3)
Supplement: Additional file 13 — RAP application directory. Sets up directory for RAP (RDF application for PHP) source code [file 1472-6947-7-3-S13.gz › rap/doc/implNotes/rdql_engine.htm]

Implementation Notes: RDQL Engine


### RDF API for PHP V0.9.1

# Implementation Notes: RDQL Engine

  

Radoslaw Oldakowski <radol@gmx.de>
  
October 2003

This document is meant for developers who are interested in further work on RDF API for PHP.
I will explain here some general concepts behind the RDQL implementation.
A thorough description of functions and variables used within the RDQL package
can be found in the detailed method reference.
For a more profound understanding of every single part of the query execution
consult the commented source code.

### Contents

|  |  |
| --- | --- |
| 1. | Introduction |
| 2. | Query Parser |
| 3. | RDQL Memory Engine |
| 4. | RDQL Database Engine |
|  |  |

### 1. Introduction

The execution of an RDQL query in RDF API for PHP V0.8 consists of four main
steps. At first, the query string is parsed into an in-memory structure represented
by a PHP array using the class `RdqlParser`. Subsequently, depending
on the storage mechanism of the model the query is performed on, this array
is passed to the corresponding engine (RdqlMemEngine for MemModels and RdqlDbEngine
for DbModels). In the second step, the query engine searches for triples matching
all patterns from the `WHERE` clause of the RDQL query and returns
a set of variable bindings. Next, this result set is filtered by evaluating
Boolean expressions specified in the `AND` clause. The last step
is the processing of the final result, i.e. creating of RAP objects (`Resource`,
`BlankNode`, `Literal`) or their plain text serialization
as values of the query variables, according to the desired format. In case
of
the former, the returned objects can be used for model update or other API
calls, or simply printed out using the method `writeQueryResultsAsHtmlTable()`
of class `RdqlEngine`.

### 2. Query Parser

Although there had already been an RDQL parser written in PHP [PHPxmlClasses]
available, it did not meet all requirements of RAP's RDQL grammar. Hence, I
have decided to develop a completely new parser from scratch.

The output of the RDQL parser used in RDF API for PHP V0.8 is a multi-dimensional
array (described in Figure 1) representing every clause
of the parsed query. In this section I will briefly explain how this structure
is generated.

`array ['selectVars'][] = ?VARNAME  
      ['sources'][]{['value']} = URI | QName  
                   {['is_qname'] = boolean}  
      ['patterns'][]['subject']['value'] = VARorURI  
                             
{['is_qname'] = boolean}  
                   
['predicate']['value'] = VARorURI  
                               
{['is_qname'] = boolean}  
                   
['object']['value'] = VARorURIorLiterl  
                            
{['is_qname'] = boolean}  
                             
['is_literal'] = boolean  
                             
['l_lang'] = string  
                             
['l_dtype'] = string  
                            
{['l_dtype_is_qname'] = boolean}  
      ['filters'][]['string'] = string  
                  
['evalFilterStr'] = string  
                  
['reqexEqExprs'][]['var'] = ?VARNAME  
                                    
['operator'] = (eq | ne)  
                                    
['regex'] = string  
                  
['strEqExprs'][]['var'] = ?VARNAME  
                                  
['operator'] = (eq | ne)  
                                  
['value'] = string  
                                  
['value_type'] = ('variable' | 'URI' | 'QName' | 'Literal')  
                                  
['value_lang'] = string  
                                  
['value_dtype'] = string  
                                 
{['value_dtype_is_qname'] = boolean}  
                  
['numExpr']['vars'][] = ?VARNAME  
     {['ns'][PREFIX] = NAMESPACE}`

|  |  |
| --- | --- |
| **Figure 1:** | An abstract description of the PHP array representing an RDQL query produced by RAP's RDQL parser ( [ ] stands for an integer index 0..N, {} are only used within the parser class and are not passed to the query engine). |

After the query string has been passed to the method `parseQuery()`
called on an object `RdqlParser`, the function `removeComments()`
is used by the parser to clear all comments. Subsequently, the string is divided
into small tokens (`tokenize()`). Each token is either a special
character (e.g. '`(`' , '`,`' , ' ') or a
character string (e.g. `?variable`) saved in the class internal variable
`$tokens` of type array. Parsing is nothing but analyzing these tokens
one by one, in the order of their appearance in the query string.

The process is initiated by the method `parseSelect()` which recognizes
tokens representing query variables from the `SELECT` clause and
stores them in the array `$parsedQuery['selectVars']` (according
to Figure1). When this function encounters a token holding
the keyword introducing a subsequent clause of the RDQL query (i.e. `FROM`
/ `SOURCE` or `WHERE`) the next method `parseFrom()`
or `parseWhere()` is called respectively.

As explained in the RDQL tutorial,
there is no need to specify the source while calling the method `rdqlQuery()`
on an active object `MemModel` or `DbModel`. However,
RAP's RdqlParser is able to parse the `FROM` clause and can be used,
for instance, together with the RDQL engine to carry out a query on multiple
documents specified by URLs, as shown in example 1.

`// Some RDQL query with two input documents  
$query = '  
SELECT *  
FROM <http://example.org/doc1.rdf>, <http://somewhere.edu/doc2.rdf>  
WHERE (?x, vcard:FN, ?z)  
USING vcard FOR <http://www.w3.org/2001/vcard-rdf/3.0#>';`

`// Create the parser object and parse the query string  
$parser = new RdqlParser();  
$parsedQuery = $parser->parseQuery($query);`

`// Create a new RDQL engine for models stored in memory   
$rdqlMemEngine = new RdqlMemEngine();`

`// Set the variable for the merged query result  
$mergedResult = array();`

`// Iterate through all sources specified in the FROM clause  
foreach ($parsedQuery['sources'] as $url) {`

   `// Load each document into a memory Model  
   $model = new MemModel();  
   $model->load($url);  
  
   // Execute the RDQL query on the Model just created  
   $queryResult = $rdqlMemEngine->queryModel($model, $parsedQuery);`

   `// Merge all query results  
   $mergedResult = array_merge($mergedResult, $queryResult);  
}`

`// Print the merged result of the query on multiple documents  
RdqlEngine::writeQueryResultAsHtmlTable($mergedResult);`

|  |  |
| --- | --- |
| **Example 1:** | RDQL query on multiple documents. |

When the parsing of the `SELECT` and `FROM` clauses is
completed, the method `parseWhere()` is called as next and all patterns
are transformed into the array `$parsedQuery['patterns']` (see Figure
1). If the object of a pattern is a literal, additional information about
its language and datatype is recorded. A token indicating the beginning of the
`AND` or the `USING` clause causes the corresponding method
`parseAnd()` or `parseUsing()` to be called.

The processing of query filters
is one of the most complex tasks the RdqlParser has to accomplish. In the `AND`
clause the tokens of each filter are combined to form a complete filter string
in order to examine the filter as a whole. At first, the parser searches inside
the string for regular
expression patterns (e.g. `?x ~~ "/\.html$/i"`) and
serializes their corresponding parts into the array described in Figure
1 (in this example: `['var'] = ?x`, `['operator'] =
'eq'`,
`['regex'] = '/\.html$/i'`). Additionally, it replaces each pattern
inside the original filter string (`['string']`) with a placeholder
(i.e. `##RegEx_$i##` where `$i` is a numerical index)
and stores this transformed string in the array (`['evalFilterStr']`).
Subsequently, the parser looks for three different kinds of string equality
expressions in the form of `?variable (eq | ne) VALUE`, where `VALUE`
can be: `?variable`, `<uri>` or `"literal"@lang^^<datatypeURI`>.
Similarly, in this case, all parts of an expression are serialized into the
array (see Figure 1) and each expression inside the
already once transformed filter (`['evalFilterStr']`) is replaced
with a placeholder (i.e. `##strEqExpr_$i##`). The processed filter
string now only contains numerical expressions and placeholders (e.g. `?x >
5 && ##strEqExpr_0##`). Finally the parser finds all variables
of the numerical expressions and stores them in the array as well.

Given this structure saved in an array, the RDL query engine will be able to
determine the Boolean value of each single *regex* and string equality
expression for a certain set of variable bindings, replace the corresponding
placeholders, and insert the values of numerical variables. An example filter
string representation for a query result to be filtered might be: (`7 >
5 && TRUE`). After having evaluated this condition, the query
engine will accept this particular set of variable bindings.

If all filters have been parsed and the query contains a `USING`
clause, the method `parseUsing()` is called. It writes all prefixes
with their corresponding namespaces into the array described in Figure
1. However, this part of the array will not be returned by the parser but
will be only used internally to replace prefixes used in the `WHERE`
and `AND` clause. All URIs contained in the in-memory representation
of the parsed query passed to the query engine will be therefore stored in full.

An advantageous and helpful feature of RAP's RdqlParser worth mentioning is
its extensive error checking. The parser is not only able to find a syntax error,
but moreover to return a message pointing to the spot in the query where the
error occurred. This is even more important since there exist several syntactically
different RDQL implementations. Example 2 presents some
RDQL clauses containing syntax errors and the corresponding error message returned
by the parser.

`SELECT ?city, ?givenN@me, ?age`

  
**Output:**

RDQL error in the SELECT clause: '?givenN@me' variable name contains
illegal characters

`WHERE (?x, ?y, "some Literal"), (<http://www.example.org,
?a, ?b)`

  
**Output:**

RDQL error in the WHERE clause: '<http://www.example.org,' - illegal
input: ',' - '>' missing

`AND ?x ~~ /\.thml$/`

  
**Output:**

RDQL error in the AND clause: '?x ~~ /\.thml$/ ' - regular expressions
must be quoted

`USING ?dc FOR <http://www.purl.org/dc/elements/1.1/>`

  
**Output:**

RDQL error in the USING clause: '?dc' - illegal input, namespace prefix
expected

|  |  |
| --- | --- |
| **Example 2:** | Error messages returned by the RDQL parser. |

### 3. RDQL Memory Engine

If an RDQL query is performed on an in-memory model, an instance of the object
`RdqlMemEngine` has to be created. The query execution is initiated
by calling the method `queryModel()` with two parameters being passed
by reference: the object `MemModel` itself and the array representation
of the query, generated by the RdqlParser.

The RdqlMemEngine begins the execution of a query with searching for tuples
matching all patterns specified in the `WHERE` clause, by calling
the corresponding method `findTuplesMatchingAllPatterns()`. This
process is divided into a sequence of two steps carried out in a loop. Initially
the result for one single pattern is found (`findTuplesMatchingOnePattern()`).
Subsequently, it is joined with the set of tuples matching the previously examined
patterns (`joinTuples()`).

The execution of both steps is rather complex. In the case of the first one,
the RdqlMemEngine analyzes all parts of the statement pattern (subject, predicate,
object) and additionally determines internal bindings (e.g. `?x, <uri>,
?x`). Given all the information, the method `findTriplesMatchingPattern()`
searches the queried model for statements fitting into the pattern also considering
the nuances of the RDQL specification. The result of this function is then converted
into an array holding variable bindings that represents a set of tuples matching
one pattern.

The join operation performed on two result sets is nothing else than a PHP
implementation of the SQL-like Inner Join. Furthermore, applying it within the
loop (compared to the approach where the results for each pattern are first
determined and then joined altogether) enables the engine to significantly reduce
memory consumption.

In this way the query engine has found all tuples matching the patterns from
the `WHERE` clause. If the RDQL query being processed additionally
contains filter expressions (`AND` clause), the result set determined
so far is passed to the method `filterTuples()`. This function, taken
one by one for each tuple, substitutes the placeholders inside the filter string
with the Boolean values of the evaluated filter *regex* and string equality
expressions; subsequently it inserts the values of the numerical variables and
evaluates the entire filter string as a PHP expression .

For a better understanding I will explain the filtering process with an example.
Consider the tuple consisting of two variable bindings: `?age = 26`,
`?name = "Radoslaw"` and the `AND` clause of
an RDQL query: `(?age < 25) && (?name eq "Radoslaw")`.
The in-memory representation of this particular filter returned by the RdqlParser
is shown in Example 3 (compare to Figure
1).

`array ['filters'][0]['string'] = '(?age < 25) && (?name
== "Radoslaw")'  
                   
['evalFilterStr'] = '(?age < 25) && (##strEqExpr_0##)'  
                   
['strEqExprs'][0]['var'] = ?name  
                                    
['operator'] = 'eq'  
                                    
['value'] = 'Radoslaw'  
                                    
['value_type'] = 'Literal'  
                                    
['value_lang'] = NULL  
                                    
['value_dtype'] = NULL  
                   
['numExpr']['vars'][0] = ?age`

|  |  |
| --- | --- |
| **Example 3:** | The in-memory representation of the AND clause:    `(?age < 30) && (?name eq "Radoslaw").` |

Given the structure described in Example 3, the query
engine at first evaluates the string equality expression by checking if the
value of the variable `?name` (i.e. `"Radoslaw"`)
is a literal with the label equal "Radoslaw" and no specified language
or datatype. In this case it is one, so the query engine replaces the placeholder
(i.e. `##strEqExpr_0##`) with `TRUE`, what results in
the filter string: `(?age < 25) && (TRUE)`. Next, the
query engine inserts the value of the numerical variable `?age` and
determines the Boolean value of the whole filter `(26 < 25) &&
(TRUE)`. In this example the output is `FALSE` ( `(26
< 25) && (TRUE) => (FALSE) && (TRUE) = FALSE` );
consequently this particular tuple will be removed from the final result.

This was just an example of a very simple filter. RAP RDQL query engine is
able to handle much more complex `AND` clauses consisting of several
filters where each of them can be built of different kinds of expressions combined
together by using logical operators. Even the process of evaluating one single
string equality expression becomes more complicated if the literal has specified
datatype and language.

In the last step, after having filtered all tuples, the query engine removes
the filter variables (which have not been specified in the `SELECT`
clause) from the result set. Since the engine has been working with RAP objects
all the time, it can now return the final query result with no further processing,
unless the parameter `$returnNodes` passed to the object `RdqlMemEngine`
has been set to `FALSE`. In this case the values of all query variables
are still to be serialized to string.

### 4. RDQL Database Engine

Similarly to the case of a MemModel, an RDQL query performed on a persistent
model (DbModel) is initiated by calling the method `queryModel()`
on an object `RdqlDbEngine` with two parameters being passed by reference:
the DbModel itself and the in-memory representation of the parsed query. Though,
from this point on the internal execution of the query takes a different course.
The searching for tuples matching all patterns from the `WHERE` clause
is this time delegated to the database management system. Moreover instead of
calling the database for the result set matching each single pattern and then
joining them in memory, the RdqlDbEngine translates almost the entire RDQL query
into an SQL statement and sends it to the database for execution. This approach
enables the machine to use the database indexing and optimization capabilities.

The algorithm used for the query rewriting is relatively simple. Every single
statement pattern is treated as a separate virtual database table (*statements*).
If the subject, predicate, or object of each pattern is a URI or a literal,
it is bound to its value. Otherwise, in the case that it is a variable, an identity
relationship between all occurrences of this variable is created. The variables
to be returned are indicated in the `SELECT` clause of the SQL statement
being generated.

Example 4 provides a sample RDQL query and its corresponding
translation into SQL produced by the RdqlDbEngine. The first two lines in the
`SELECT` clause of the SQL statement describe the variable `?someone`.
Due to its mapping to the subject (of the first triple), it can either be a
resource or a Blank Node. To find this out we need to know the value of the
corresponding table column (*subject\_is*). In the case of the next variable
`?name` mapped to the object of the second triple, it can additionally
be a literal as well, so that the information about its language and datatype
must also be returned. The `FROM` clause indicates three virtual
tables (*statements*) each representing a pattern of the RDQL query. In
the `WHERE` clause of the SQL statement there are constraints specified
for every part of a triple. Respectively, the subject in the first table (`s1.subject`)
representing the variable `?someone` is bound to the subject in the
second table (`s2.subject`) holding the same variable; the predicate
(`s1.predicate`) and object (`s1.object`) are equated
to the string they are supposed to match. Since RAP supports storing of multiple
models in a database, each pattern has to be bound to the `modelID`
of the model the query is performed on. Note that variables `?name`
and `?y` occur only once so that they are not constrained by any
binding and can therefore match anything. Furthermore, the value of the variable
`?name` must be returned by the database even if it is not specified
in the `SELECT` clause of the RDQL query. It will be needed to evaluate
the filter expressions (`?name EQ "Brian" || ?name EQ "Carl"`),
which will be carried out in main memory. However, this variable will only be
used internally within the RdqlQueryEngine and will not be included in the returned
final result set.

**RDQL query:**

`SELECT ?someone, ?name  
WHERE (?someone, <http://ex.org/vocab/isFirendOf>, <http://ex.org/John>),  
      (?someone, <http://ex.org/vocab/name>, ?name),  
      (?someone, ?y, "35"^^http://www.w3.org/2001/XMLSchema#integer)  
AND (?name EQ "Brian" || ?name EQ "Carl")`

  
**The corresponding SQL-Statement:**

`SELECT s1.subject as _someone ,   
       s1.subject_is ,`

       `s2.object as _name ,   
       s2.object_is ,   
       s2.l_language ,   
       s2.l_datatype`

`FROM statements s1 ,   
     statements s2 ,   
     statements s3`

`WHERE s1.modelID=39   
AND s1.subject=s2.subject   
AND s1.predicate='http://ex.org/vocab/isFirendOf'   
AND s1.object='http://ex.org/John'`

`AND s2.modelID=39   
AND s2.subject=s3.subject  
AND s2.predicate='http://ex.org/vocab/name'`

`AND s3.modelID=39   
AND s3.object='35'   
AND s3.object_is='l'   
AND s3.l_datatype='http://www.w3.org/2001/XMLSchema#integer`

|  |  |
| --- | --- |
| **Example 4:** | An example query and its corresponding SQL translation. |

The technique used for the query rewriting is very similar to Matt Biddulp's
algorithm for translating rdfDB-style queries into SQL ([Biddulph
2002], [Miller 2002]).
It has been extended in order to
additionally retrieve variable bindings for filter variables used in the `AND`
clause of an RDQL query and adapted to RAP's database layout.

RdqlDbEngine uses ADOdb abstraction
layer for SQL query execution. Thus, the result of every successful database
query is returned in the form of an object `ADORecordSet` holding
the found database records in the form of an array, where the values of each
single record field are associated with integer keys. However, because of the
fact that SQL statements are generated dynamically, the mapping of these indexes
to the query variables is different for every RDQL query. Fortunately, RdqlDbEngine
provides a solution to this problem: while generating an SQL statement it associates
each RDQL query variable with the corresponding index of the `ADORecordSet->fields`
array. This information is stored in the class private variable `$rsIndexes`.
This approach allows the query engine to emulate indexing by variable name while
retrieving SQL query results.

The filtering of the tuples matching all patterns specified in the `WHERE`
clause of the RDQL query is carried out in memory. The process is very similar
to the one performed by the RdqlMemEngine with the major difference being that
the RdqlDbEngine does not operate on RAP objects (`Resource`, `Literal`,
`BlankNode`), but on the object `ADORecordSet` returned
by the database. Finally in the last step the values of the variable bindings
have to be converted either to RAP objects or to their string serialization
according to the desired format of the RDQL query result set.
